# Abnormal large‐scale brain functional network dynamics in social anxiety disorder

**DOI:** 10.1111/cns.14904

**Published:** 2024-08-06

**Authors:** Xun Zhang, Baolin Wu, Xun Yang, Graham J. Kemp, Song Wang, Qiyong Gong

**Affiliations:** ^1^ Department of Radiology and Huaxi MR Research Center (HMRRC), Functional and Molecular Imaging Key Laboratory of Sichuan Province, West China Hospital Sichuan University Chengdu China; ^2^ Research Unit of Psychoradiology Chinese Academy of Medical Sciences Chengdu China; ^3^ School of Public Affairs Chongqing University Chongqing China; ^4^ Liverpool Magnetic Resonance Imaging Centre (LiMRIC) and Institute of Life Course and Medical Sciences University of Liverpool Liverpool UK; ^5^ Department of Radiology West China Xiamen Hospital of Sichuan University Xiamen China

**Keywords:** dynamic functional connectivity, independent component analysis, magnetic resonance imaging, psychoradiology, resting‐state networks, social anxiety disorder

## Abstract

**Aims:**

Although static abnormalities of functional brain networks have been observed in patients with social anxiety disorder (SAD), the brain connectome dynamics at the macroscale network level remain obscure. We therefore used a multivariate data‐driven method to search for dynamic functional network connectivity (dFNC) alterations in SAD.

**Methods:**

We conducted spatial independent component analysis, and used a sliding‐window approach with a k‐means clustering algorithm, to characterize the recurring states of brain resting‐state networks; then state transition metrics and FNC strength in the different states were compared between SAD patients and healthy controls (HC), and the relationship to SAD clinical characteristics was explored.

**Results:**

Four distinct recurring states were identified. Compared with HC, SAD patients demonstrated higher fractional windows and mean dwelling time in the highest‐frequency State 3, representing “widely weaker” FNC, but lower in States 2 and 4, representing “locally stronger” and “widely stronger” FNC, respectively. In State 1, representing “widely moderate” FNC, SAD patients showed decreased FNC mainly between the default mode network and the attention and perceptual networks. Some aberrant dFNC signatures correlated with illness duration.

**Conclusion:**

These aberrant patterns of brain functional synchronization dynamics among large‐scale resting‐state networks may provide new insights into the neuro‐functional underpinnings of SAD.

## Graphical abstract

Four distinct recurring states were identified, including (in order of decreasing frequency) state with “widely weak” FNC, state with “widely moderate” FNC, state with “locally strong” FNC (mainly involving perceptual networks), and state with “widely strong” FNC. SAD patients significantly spent more time in a “weakly connected” state and less time in “strongly connected” states and demonstrated decreased FNC mainly within/between the default mode network, the attention network, and perceptual networks.

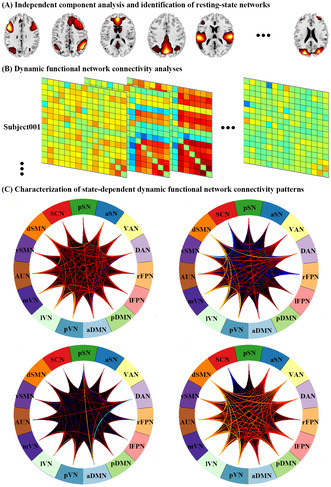

## 1 INTRODUCTION

Social anxiety disorder (SAD) is a debilitating and disabling psychiatric disorder, characterized by marked and disproportionate anxiety and fear of potential negative evaluations by others, leading to avoidance behavior in social situations.^1^ Its lifetime prevalence is approximately 13%,^2^ and it is often comorbid with other psychopathologies such as other types of anxiety disorders, major depressive disorder, and substance abuse.^3^ SAD typically has a chronic course, often poorly treated,^4^ leading to potentially severe emotional, cognitive, and behavioral dysfunction.^5^ Unfortunately, the pathophysiology of SAD is still not well understood, although the noninvasive approaches of magnetic resonance imaging (MRI), particularly functional MRI (fMRI), have proved informative.^6^, ^7^ The relatively consistent fMRI findings on SAD are hyper‐activation of the “fear circuitry” (i.e., the fronto‐limbic circuitry) mainly including the prefrontal cortex, insula, anterior cingulate cortex, and amygdala,^8^ with more recent reports of hyper‐activation of medial parietal and occipital areas and hypo‐connectivity of parietal, limbic and executive network regions.^9^ Such findings inform a model of SAD pathophysiology that abnormal bottom–up responses and top–down regulation may trigger the characteristic emotional hyperarousal and dysfunctional cognitive control.^9^, ^10^, ^11^, ^12^

Notably, most of this evidence is based on task‐evoked fMRI, prompting the question of whether similar patterns can be detected using resting‐state fMRI (rs‐fMRI), which avoids potential confounding effects of task or stimulus.^13^, ^14^, ^15^, ^16^ Given the brain is increasingly recognized as a system of information‐interacting networks,^17^ using rs‐fMRI data to probe functional connectivity (FC), defined as the temporal coherence of low frequency oscillations of spatially distant brain regions,^18^ can provide particular insights into macroscale spatiotemporal organization and functional interactions of brain networks.^19^ There are two main approaches to FC: calculations based on an atlas or regions of interest (ROI), and data‐driven methods such as independent component analysis (ICA), which enables a whole‐brain analysis to identify large‐scale spatially independent functional networks that are temporally coherent. ICA is largely immune to template/ROI definitional differences and inter‐subject spatial variability^20^, ^21^ and can investigate FC at the large‐scale network level and assess their fundamental network architecture without restricting the analyses scope to a specified set of regional connectivities; this is referred to as functional network connectivity (FNC).^22^ Indeed, ICA is proving a powerful approach to explore brain normal or abnormal functional synchronization at the macroscale network level,^23^, ^24^, ^25^, ^26^ but has not been much used in SAD. The only existing research indicated that FNC abnormalities in the high‐order cognitive networks and primary systems may be involved in SAD patients.^27^, ^28^, ^29^, ^30^ Especially, our recent work found that SAD patients had widespread intra‐network connectivities alterations in the default mode network (DMN), the subcortical network (SCN), and the perceptual system (i.e., visual, auditory, and sensorimotor networks), and aberrant inter‐network connectivities among these primary and high‐order networks.^27^

However, like most conventional rs‐fMRI research, those studies made the implicit assumption that brain activity and FC remain stationary during the entire MRI scanning period. However, given the increasing evidence that even the “resting” brain is a highly nonstationary system with rapidly fluctuating functional activities and interactions, valuable information may be lost in a purely static rs‐fMRI analysis.^31^, ^32^, ^33^ This may explain some of the heterogeneity of resting‐state FC findings: the static approach conveniently simplifies the brain activity/connectivity analysis by averaging across different dynamic activity modes,^34^ but at the cost of missing important brain temporal patterns (i.e., dynamics) which may provide indispensable and complementary insights into the neurobiology and index changes in macroscopic neural activity patterns encoding critical aspects of cognition and behavior.^22^, ^35^ Notably, examining brain dynamics in the resting state is of vast importance to probe the brain intrinsic mechanisms as the mental activity is not directly constrained and modulated by task performances.^32^ It has long been recognized that subjects are freely implicated in various types of mental activity at rest, of which the predominance has an impact on the FC and modular organization throughout the brain.^36^ Previous work has found that dynamic FC (dFC) patterns were related to cognitive and affective processes^37^, ^38^ and altered in several neuropsychiatric disorders^34^, ^39^, ^40^, ^41^, ^42^; furthermore, dynamic FNC (dFNC) features significantly outperformed static FNC in the classification of schizophrenia and bipolar disorder patients, indicating the dFNC may be a more sensitive marker.^43^ However, to our knowledge, there have been no studies in SAD of the aberrant dFNC patterns of large‐scale brain networks and their potential clinical relevance.

We therefore set out, in a relatively large homogenous sample of SAD patients, to perform data‐driven ICA with rs‐fMRI data to characterize a set of resting‐state networks (RSNs), and to adopt a sliding‐window approach with a k‐means clustering algorithm, a widely used method for quantifying brain dynamics,^32^ to comprehensively identify the variation patterns of intrinsic inter‐network dFNC. For the dynamic states thus identified, we compared the transition properties and corresponding FNC strength between SAD patients and healthy controls (HC) and further investigated the associations with clinical features. Given there is deficient evidence on brain dynamics in SAD and the current study is more of exploratory, no specific hypothesis is made to be tested.

## 2 METHODS

### 2.1 Subjects

At the Mental Health Center of the West China Hospital of Sichuan University, 49 right‐handed SAD patients with no psychiatric comorbidity were recruited,^44^, ^45^ the diagnosis of SAD being made by two experienced clinical psychiatrists using the Structured Clinical Interview for Diagnostic and Statistical Manual of Mental Disorders, Fourth Edition (Patient Edition) (SCID). According to power analysis using G Power software,^46^ a medium‐sized effect with adequate statistical power by independent‐sample *t*‐test (Cohen's *d* = 0.5, *α* = 0.05, 1‐β = 0.8) requires at least 102 subjects. Consequently, for comparison, we recruited 53 demographically matched (i.e., sex, age, and handedness) HC from the local community, who were free from any neuropsychiatric disease verified by the SCID‐non‐patient version. The exclusion criteria for all subjects were: comorbidity with other psychiatric disorders; current psychological or psychopharmacological treatment; history of substance abuse or dependence; learning or neurodevelopmental illness; history of head injury or neurosurgery, and significant systemic or neurologic illness; family history of mental disorders; age under 18 or over 60 years; current pregnancy, claustrophobia, or other MRI contraindications.

Illness duration was determined as the period between the first reported/observed changes in psychological/behavior state and the moment of participation in this study,^47^ information being verified by patients, family members, and medical records. We used the self‐reported Liebowitz Social Anxiety Scale (LSAS),^48^ common in SAD studies, to assess social anxiety levels of all subjects; the LSAS includes scores for social avoidance factor (LSASA) and fear factor (LSASF), their sum being the total score (LSAST). Good validity and reliability are demonstrated in the LSAS of Chinese populations.^49^, ^50^

In accordance with relevant national and institutional policy and the Helsinki Declaration of 1975, this study was approved by the Medical Research Ethics Committee of West China Hospital of Sichuan University. Fully‐informed written consent was obtained from all participants.

### 2.2 Image acquisition and preprocessing

#### 2.2.1 Image acquisition

Whole‐brain high‐resolution three‐dimensional T1‐weighted images and rs‐fMRI data were obtained using a 3.0 T MR scanner (Siemens Trio, Erlangen, Germany) with a 12‐channel head coil. Subjects were instructed to lie still with eyes closed, relaxed but awake during the scans. We provided earplugs to reduce the effects of scanner noise and foam pads to minimize head motion. A spoiled gradient‐recalled sequence was used to acquire three‐dimensional T1‐weighted images: inversion time (TI)/repetition time (TR)/echo time (TE) 900 ms/1900 ms/2.26 ms, flip angle 9°, slice thickness 1 mm, data matrix 256 × 256, 176 sagittal slices. A gradient echo‐planar imaging sequence was utilized to collect rs‐fMRI data: TR/TE 2000 ms/30 ms, flip angle 90°, acquisition matrix 64 × 64, field of view 240 × 240 mm^2^, thickness 5.0 mm without gap, 205 volumes. An experienced neuroradiologist inspected all MRI data so as to exclude subjects with visible artifacts or lesions.

#### 2.2.2 Image preprocessing

Using the toolbox for Data Processing & Analysis of Brain Imaging (DPABI, http://rfmri.org/DPABI),^51^ preprocessing of the rs‐fMRI data included: removal of the first 10 volumes; slice timing correction; realignment and head motion correction (3 SAD and 1 HC were excluded for head motion >2.5 mm or 2.5°), with calculation of frame‐wise displacement (FD) to characterize head motion for subsequent analyses; co‐registration of individual structural images to the mean of functional images, and segmentation of transformed structural images; spatial normalization to Montreal Neurological Institute space using the Diffeomorphic Anatomical Registration Through Exponentiated Lie algebra tool^52^; resampling functional images into 3 × 3 × 3 mm^3^ and spatial smoothing with an 8 mm full‐width at half‐maximum Gaussian kernel to improve the signal‐to‐noise ratio.

### 2.3 Independent component analysis and identification of resting‐state networks

After preprocessing, we conducted spatial group ICA (GICA) using Group ICA Of fMRI Toolbox (GIFT) software (http://mialab.mrn.org/software/gift/) in MATLAB R2014a (The MathWorks, Inc). GICA automatically estimates the number of independent components (ICs) via decomposing the fMRI data into spatially ICs accompanied by a unique time course profile.^53^ Principal component analysis was first conducted to decompose subject‐specific rs‐fMRI data. Next, the InfoMax algorithm was used to the reduced dataset of all subjects which was concatenated over time, the concatenated subject‐reduced data being decomposed into 24 ICs. To maximize reliability and stability, this was repeated 20 times using ICASSO (http://research.ics.tkk.fi/ica/icasso/), the most central run being selected for further analyses.^54^ Finally, subject‐specific spatial ICs maps and time courses were produced using a GICA back‐reconstruction approach.^55^ To identify which ICs represented meaningful RSNs, we combined visual inspection (e.g., peak activations of spatial maps in gray matter with minimal overlap with known ventricles, vessels, motion, and susceptibility artifacts, as well as the dominant low‐frequency power of time courses)^22^ with the examination of the spatial correlation with RSNs reference templates via the “sort components” tool of the GIFT toolbox (greater regression coefficient suggesting more spatial similarity to the templates).^30^, ^56^ We thus identified 15 meaningful RSNs (out of the 24 ICs), including left and right frontoparietal network (lFPN and rFPN); anterior and posterior default mode network (aDMN and pDMN); anterior and posterior salience network (aSN and pSN); dorsal and ventral attention network (DAN and VAN); SCN; dorsal and ventral sensorimotor network (dSMN and vSMN); auditory network (AUN); lateral, medial, and posterior visual network (lVN, mVN, and pVN) (Figure S1).

### 2.4 Dynamic functional network connectivity analyses

#### 2.4.1 Computation of dFNC

The temporal dFNC toolbox in GIFT was used to examine dFNC. Before the computation of windowed matrixes, additional postprocessing was conducted on time courses of identified RSNs with some steps of linear, quadratic, and cubic de‐trending, de‐spiking detected outliers, and low‐pass filter with 0.15 Hz cut‐off.^56^ Then a sliding‐window method was used to probe the time‐varying alterations of FNC among the identified 15 RSNs. Briefly, we chose a 22‐TR window (44 s) in view of the evidence that characteristics of resting‐state FNC fluctuations can be well delineated based on the time windows of 30–60 s.^32^ A Gaussian (*σ* = 3 TRs) function was used to generate a tapered window, sliding one TR step‐wise along the whole scan time series, thus producing an individual symmetric 15 × 15 inter‐network FNC matrix by Pearson's correlations in each sliding window. The L1 norm penalty was performed in the LASSO framework (100 repetitions) to promote sparsity in estimations.^57^ Finally, variance from the windowed FNC correlations related to age, sex, and mean FD was removed for each subject, and the FNC matrixes were z‐normalized with Fisher's r‐to‐z transformation.

#### 2.4.2 Identification of dFNC states

To assess recurring dFNC patterns (i.e., connectivity states) with respect to their frequency and structure, the k‐means clustering algorithm was applied to all windowed 15 × 15 FNC matrixes for all subjects, where the Manhattan city distance was adopted to evaluate their similarity.^58^ For each k value, the k‐means clustering algorithm was iterated 500 times to minimize the potential bias of the initial random selection of cluster centroids. The elbow criterion of the cluster validity index was adopted to identify the optimal number of clusters (*k* = 4 in the current study).^22^ The best run across the 500 iterations (*k* = 4) was kept for further analysis, the resulting 4 cluster centroids representing 4 recurring dFNC states. We also measured each group‐specific state centroid by calculating an average of all subject‐specific centroids for each group to characterize group‐specific dFNC state patterns (note that this does not entail that every subject possesses all 4 dFNC states).

### 2.5 Characterization of state‐dependent dFNC patterns

#### 2.5.1 Temporal properties of state‐dependent dFNC patterns

To further characterize the temporal features of dFNC states, we computed 3 commonly used state transition metrics: fractional window/time, the proportion of time that a subject spends in a specified state; mean dwell time, the average time that a subject stays in a given state before switching to another; and number of transitions, the total number that a subject switches from one state to another during the whole scanning course. The Shapiro–Wilk test was used to assess the normality of all continuous variables, following which the independent‐sample t‐test was used for normally distributed data, and the nonparametric permutation test for nonnormal data. Accordingly, nonparametric permutation tests (10,000 iterations) were performed to compare these state transition metrics between SAD and HC, with age, sex, and mean FD as covariates. The false discovery rate (FDR) approach was performed for multiple comparisons correction with *p* < 0.05 as a significance threshold.^59^

#### 2.5.2 Connections strength of state‐dependent dFNC patterns

Separately, FNC strength alterations in each state were assessed using network‐based statistic (NBS) analysis; this network graph analog of cluster‐based thresholding of statistical parametric maps, based on the hypothesis that disrupted connections tend to compose distributed subnetworks which may be more likely to reflect the underlying neurobiological alterations than isolated connections, controls the family wise error (FWE) rate well for multiple comparisons.^60^ Briefly, for each subject, all the FNC matrixes belonging to one state were first collected to compute a median matrix. Then suprathreshold connections (*p* < 0.005 for independent‐sample t‐test design) were collected to identify interconnected components (i.e., subnetworks) and their size (i.e., the number of connections). Finally, to assess the significance of each connected component, nonparametric permutation tests (5000 permutations) were used to derive the empirical null distribution of the largest connected components. Specifically, for each permutation, subjects were randomly exchanged between the SAD and HC groups, and the size of the largest connected component in the randomized data was recorded. FWE‐corrected *p* for a component of size N present in the real grouping (SAD vs HC) was calculated as the percentage of the 5000 permutations for which the largest connected component was larger than or equal to N.^61^

#### 2.5.3 Clinical relevance of state‐dependent dFNC patterns

We conducted an exploratory analysis of the relationships between the dFNC state transition abnormalities and clinical features (i.e., LSASA, LSASF, and illness duration) in the SAD cohort, using Spearman's correlation analysis (given the nonnormality of dynamic indexes).^62^ *p* < 0.05 was considered significant in this exploratory analysis for descriptive and heuristic purposes.

### 2.6 Validation analyses

To verify the main findings on dFNC states from k‐means clustering with a sliding‐window length of 22‐TR, we repeated the analysis with two other window lengths (20‐TR and 30‐TR), then computed Pearson's correlation coefficients between the resulting cluster centroids, the states with the highest correlation coefficient being considered the same state. We also used nonparametric permutation tests (10,000 iterations) to compare the between‐group differences of the fractional time and mean dwell time for each state, in addition to the number of transitions from two other window lengths.

## 3 RESULTS

### 3.1 Demographic and clinical characteristics

After image preprocessing, data from 46 SAD patients and 52 HC were included for final analysis. The groups did not differ significantly regarding age and sex, but significantly higher LSAS scores were observed in SAD patients (Table 1). There was no significant between‐group difference in mean FD (*t* = 0.519, *p* = 0.605).

**TABLE 1:** Demographics and clinical characteristics of participants.

Characteristics	SAD (*N* = 46)	HC (*N* = 52)	*p*‐value
Sex (male/female)	28/18	30/22	0.749^a^
Age (years)	24.8 ± 5.3	23.3 ± 3.1	0.07^b^
Illness duration (years)	7.1 ± 4.2	–	–
LSAST	65.2 ± 23.1	18.6 ± 8.5	<0.001^b^
LSASF	32.6 ± 11.6	10.3 ± 5.3	<0.001^b^
LSASA	32.6 ± 12.8	8.3 ± 6.1	<0.001^b^

*Note*: Continuous variables are presented as mean ± standard deviation.

Abbreviations: HC, healthy controls; LSAST, LSASF, and LSASA, total score and fear and avoidance factor scores on the Liebowitz Social Anxiety Scale (LSAS); SAD, social anxiety disorder.

^a^ *p* value obtained using a chi‐square test.

^b^ *p* value obtained using an independent‐sample *t*‐test.

### 3.2 General characteristics of dFNC patterns

**FIGURE 1:**
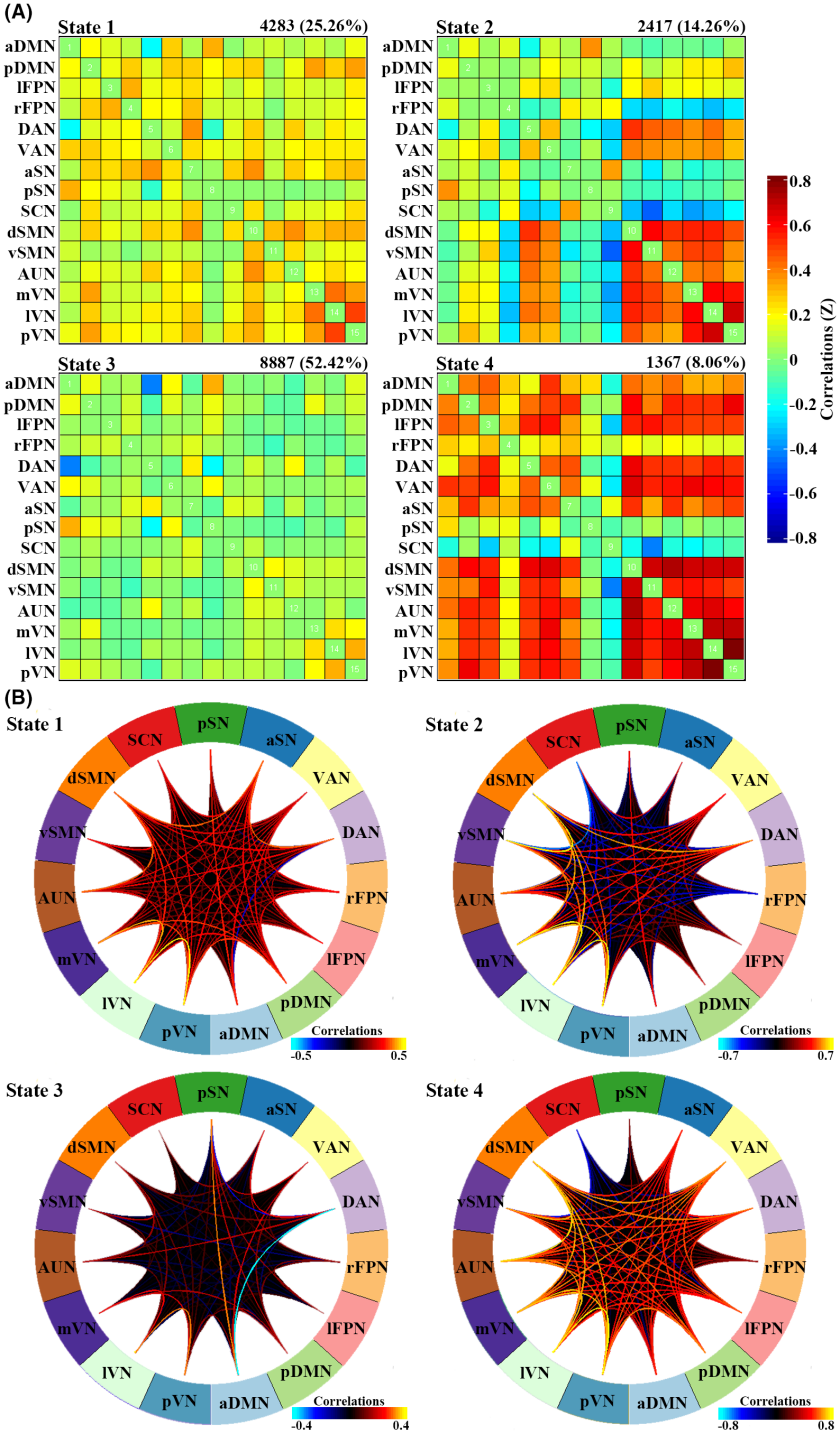
Cluster centroids and corresponding functional connectograms for each state over the whole sample (window size: 22‐TR). (A) Cluster centroids for each of the 4 dynamic functional network connectivity states. These matrixes show correlations (warmer colors representing positive and colder colors negative) between the 15 identified networks: At the top right corner of each matrix is the total number of temporal occurrence (and the percentage) of corresponding states. (B) Functional connectograms for each of the 4 states. aDMN, anterior default mode network; aSN, anterior salience network; AUN, auditory network; DAN, dorsal attention network; dSMN, dorsal sensorimotor network; lFPN, left frontoparietal network; lVN, lateral visual network; mVN, medial visual network; pDMN, posterior default mode network; pSN, posterior salience network; pVN, posterior visual network; rFPN, right frontoparietal network; SCN, subcortical network; VAN, ventral attention network; vSMN, ventral sensorimotor network.

**FIGURE 2:**
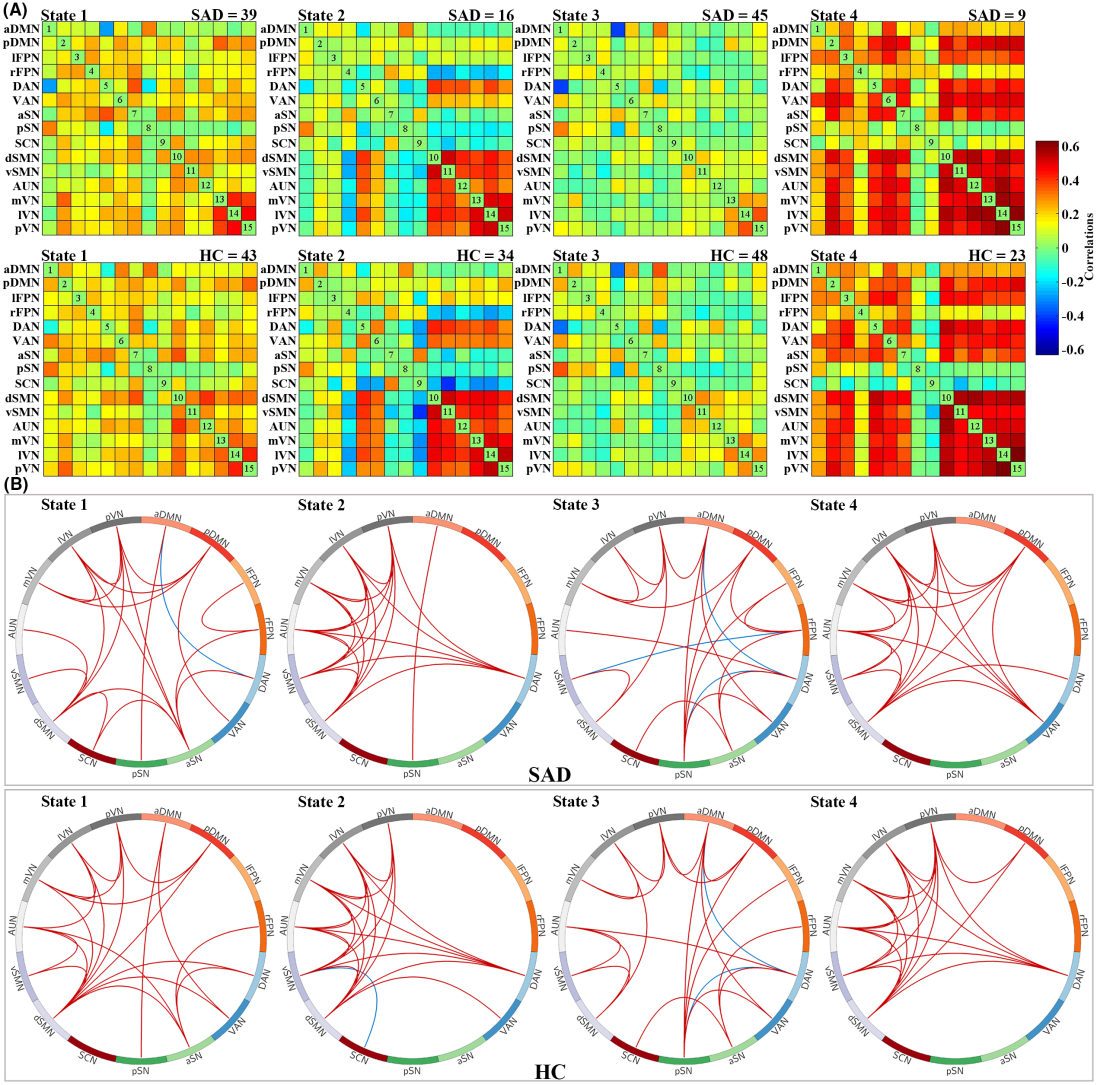
Group‐specific cluster centroids and corresponding connections with the top 20% in functional network connectivity strength for each state (window size: 22‐TR). (A) Group‐specific cluster centroids for each dynamic functional network connectivity state. At the top right corner of each matrix is the number of subjects (top row SAD patients, second row HC) possessing the corresponding states. (B) Group‐specific connections with the top 20% in functional network connectivity strength of corresponding cluster centroids for each state (top row SAD patients, second row HC). aDMN, anterior default mode network; aSN, anterior salience network; AUN, auditory network; DAN, dorsal attention network; dSMN, dorsal sensorimotor network; HC, healthy controls; lFPN, left frontoparietal network; lVN, lateral visual network; mVN, medial visual network; pDMN, posterior default mode network; pSN, posterior salience network; pVN, posterior visual network; rFPN, right frontoparietal network; SAD, social anxiety disorder; SCN, subcortical network; VAN, ventral attention network; vSMN, ventral sensorimotor network.

Using the k‐means clustering algorithm, we identified four dFNC states that recurred throughout individual scans and across participants. State 3 occurred most frequently (52.42%), State 1 at a moderate frequency (25.26%), State 2 at a lower frequency (14.26%), and State 4 at the lowest frequency (8.06%). Figure 1 shows the correlation matrix of centroids and the corresponding functional connectogram for each of the 4 states over the whole sample. Figure 2 shows group‐specific correlation matrix of centroids and corresponding connections with the top 20% in FNC strength for each of the 4 states. As shown in Figures 1 and 2, State 3 was characterized by “widely weaker” FNC, State 1 demonstrated “widely moderate” FNC, State 2 showed “locally stronger” FNC mainly involving perceptual networks (i.e., visual, auditory, and sensorimotor networks), and State 4 featured “widely stronger” FNC, especially among perceptual networks and pDMN, lFPN, and VAN. Accordingly, the averaged FNC for a given network with all others was strongest in State 4, weakest in State 3, and moderate in States 1 and 2 (Figure S2).

### 3.3 Group differences in temporal transition vectors and strength of dFNC states

**FIGURE 3:**
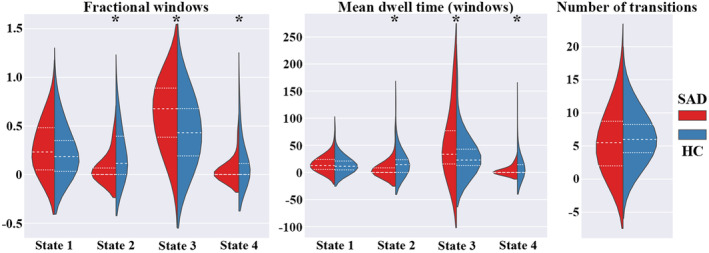
Between‐group differences of temporal transition vectors for state‐dependent dynamic functional network connectivity patterns in SAD patients and HC (window size: 22‐TR). The figure shows the distribution of each of the 3 temporal vectors across the 4 states in the two groups. *p < 0.05 for group comparisons between SAD patients and HC. HC, healthy controls; SAD, social anxiety disorder.

Figure 3 shows between‐group differences of temporal transition vectors for state‐dependent dFNC patterns. Although both groups had similar cluster centroids of dFNC states, SAD patients had significantly higher fractional time and mean dwelling time in State 3, but lower in States 2 and 4. There were no significant between‐group differences in the number of transitions.

**FIGURE 4:**
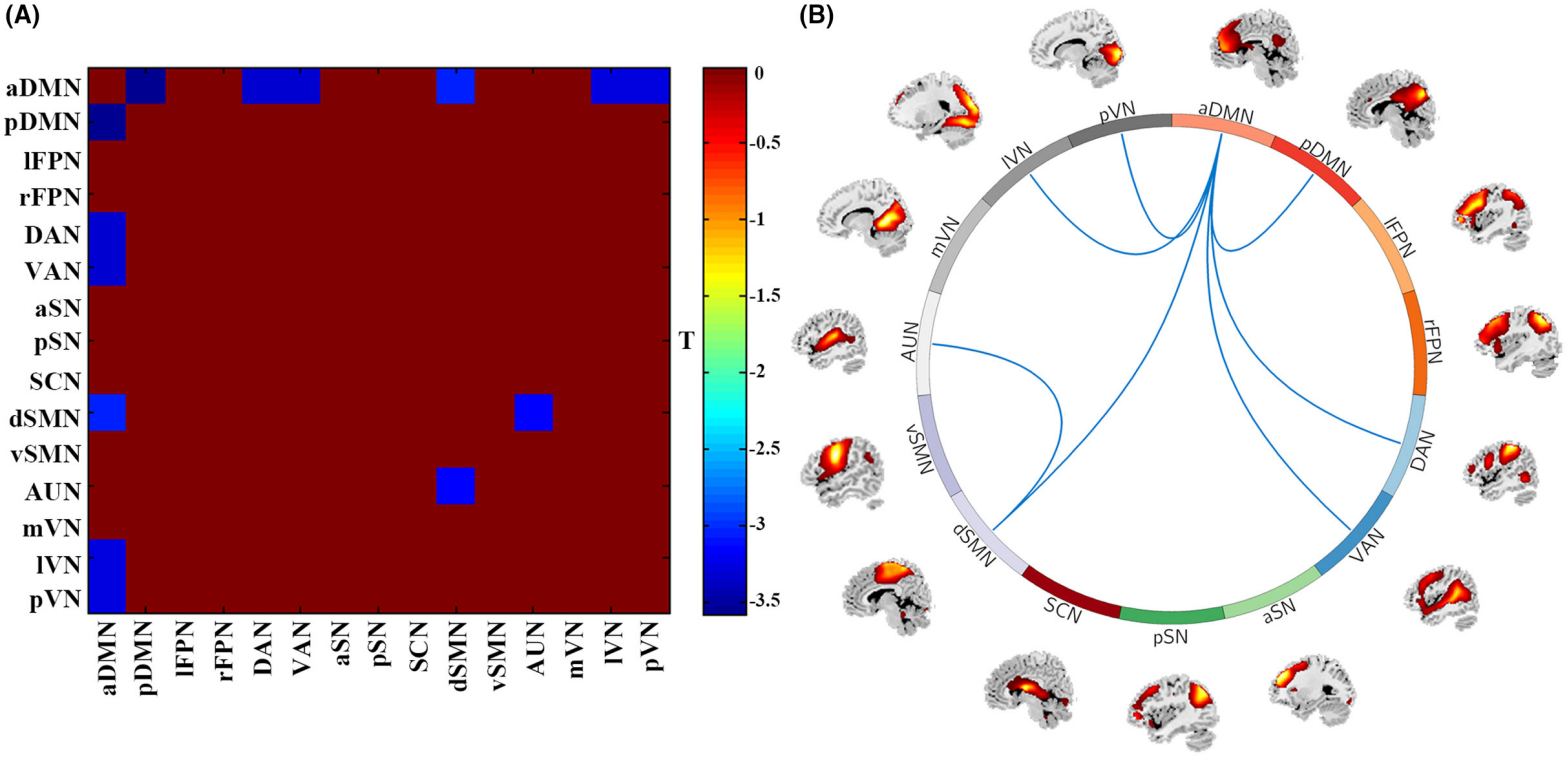
Between‐group differences of FNC strength for each state in SAD patients and HC (window size: 22‐TR). (A) The matrix of between‐group comparisons indicates the significant connections in SAD patients compared to HC. Warmer colors represent increased inter‐network FNC, cooler colors decreased inter‐network FNC in SAD. (B) Decreased inter‐network FNC in SAD patients compared to HC. aDMN, anterior default mode network; aSN, anterior salience network; AUN, auditory network; DAN, dorsal attention network; dSMN, dorsal sensorimotor network; FNC, functional network connectivity; HC, healthy controls; lFPN, left frontoparietal network; lVN, lateral visual network; mVN, medial visual network; pDMN, posterior default mode network; pSN, posterior salience network; pVN, posterior visual network; rFPN, right frontoparietal network; SAD, social anxiety disorder; SCN, subcortical network; VAN, ventral attention network; vSMN, ventral sensorimotor network.

Figure 4 shows between‐group differences in FNC strength. Compared to HC, SAD patients had decreased FNC between aDMN with pDMN, DAN, VAN, dSMN, lVN, pVN, and between dSMN with AUN in State 1.

### 3.4 Clinical correlates of dFNC patterns

Illness duration was significantly positively correlated with fractional time in State 2 and State 4 (*r* = 0.313, *p* = 0.034; *r* = 0.291, *p* = 0.049, respectively). No significant relationship between other dynamic metrics and clinical characteristics in the SAD cohort.

### 3.5 Validation analyses

As for validation analysis with the window size of 20‐TR, similar features of dFNC states (i.e., significant correlations of centroid matrixes) were observed between State 1 at 20‐TR and State 1 at 22‐TR (*r* = 0.9995, *p* < 0.001), State 4 at 20‐TR and State 2 at 22‐TR (*r* = 0.9998, *p* < 0.001), State 3 at 20‐TR and State 3 at 22‐TR (*r* = 0.9998, *p* < 0.001), and State 2 at 20‐TR and State 4 at 22‐TR (*r* = 0.9997, *p* < 0.001). In validation analysis with the window size of 30‐TR, similar features of dFNC states were observed between State 1 at 30‐TR and State 1 at 22‐TR (*r* = 0.9973, *p* < 0.001), State 3 at 30‐TR and State 2 at 22‐TR (*r* = 0.9972, *p* < 0.001), State 4 at 30‐TR and State 3 at 22‐TR (*r* = 0.9988, *p* < 0.001), and State 2 at 30‐TR and State 4 at 22‐TR (*r* = 0.9918, *p* < 0.001). Besides, the state transition metrics showed the same significant between‐group differences at the different window sizes (20‐TR and 30‐TR) as the main results with 22‐TR (Figures S3 and S4).

## 4 DISCUSSION

To the best of our knowledge, this is the first study to investigate dFNC patterns of large‐scale brain networks in a relatively large homogeneous sample of SAD patients. We found that both SAD patients and HC had similar dFNC states, including (in order of decreasing frequency) State 3 with “widely weak” FNC, State 1 with “widely moderate” FNC, State 2 with “locally strong” FNC (mainly involving perceptual networks), and State 4 with “widely strong” FNC; however, SAD patients spent significantly more time in a “weakly connected” state (State 3) and less time in “strongly connected” states (States 2 and 4). Compared to HC, SAD patients showed decreased FNC between aDMN with pDMN, DAN, VAN, dSMN, lVN, pVN, and between dSMN with AUN. Abnormal dFNC patterns correlated with SAD duration, suggesting pathophysiological relevance. These findings may provide new insights into the neurophysiological mechanisms underlying SAD.

### 4.1 Aberrant temporal transition properties of state‐dependent dFNC patterns in SAD

The clustering analysis identified 4 discrete recurring dFNC states signifying discrete brain states during scanning. Compared with HC, SAD patients had more occurrences of, and spent more time in, a weakly connected state, possibly reflecting baseline neuronal activity in the resting‐state brain,^22^ and less time in a strongly connected state. This abnormal temporal distribution of brain dynamic states in SAD patients may imply aberrant information transfer among RSNs, i.e., abnormal dynamic rhythm of global integration of brain networks.^32^, ^62^ To some extent, this may support the idea of SAD as a “disconnected disorder” in which emotional dysregulation and cognitive impairments originate from the unstable neuronal dynamics of macroscale networks.^63^, ^64^, ^65^

Not only did SAD patients spend more time in a weakly connected dFNC state but they also more quickly switched back away from the strongly‐connected state when it was engaged. These results offer additional evidence that brain FNC is indeed highly dynamic, representing the flexibility of functional synchronization among different RSNs.^35^ They also suggest that previous observations about dysconnectivity (especially hypo‐connectivity) in SAD are partly a result of more frequent residence in an “idling” state (e.g., State 3) instead of stronger connectivity in task‐active networks, as SAD patients were more likely to transition from strongly connected to other states.^27^ Both phenomena, fewer switches into the strongly connected state and faster exits from that state once engaged, may represent an impaired ability to transition into the more energy‐demanding strongly connected states required by task‐active networks, possibly contributing to the emotional, cognitive, and behavioral impairments in SAD.^66^ Intriguingly, patients with other psychiatric disorders such as schizophrenia have been reported to spend more time in a weakly connected state.^34^ It has been suggested that the weakly connected dFNC state relates to self‐focused thinking^67^; self‐focused attention bias, rumination, and negative emotional experiences are prominent in SAD.^68^, ^69^ In this sense, it could be speculated that the reason why SAD patients spend more time in the weakly connected state might be that most of their time was implicated in self‐focused thinking during the resting‐state.

### 4.2 State‐dependent inter‐network dFNC abnormalities in SAD

#### 4.2.1 Intra‐DMN FNC

The DMN is critical for social, affective, and introspective processes such as self/other‐reference processing, autobiographical memory, analyzing others' mental states, and recollection of experiences.^70^ The aDMN and pDMN subsystems, of which the medial PFC and posterior cingulate cortex are the respective hubs, interact well with the core system^71^; generally, the aDMN is responsible for self/other‐referential evaluations, while the pDMN is involved in autobiographical/episodic memory retrieval and scene construction.^72^ An optimal balance and interaction between aDMN and pDMN is critical for high‐level cognitive and affective processes,^73^ and aberrant aDMN/pDMN coupling has been observed in some neuropsychiatric disorders.^74^, ^75^, ^76^ Generally, dysfunction of DMN is closely related to functional cognitive models of disturbed self‐evaluative and referential processes, such as postevent rumination, maladaptive self‐focused attention, excessive focus and unreasonable conjecture on others' intentions.^63^ In this sense, our observation of hypo‐connectivity between aDMN and pDMN may reflect aberrant functional coupling, underlying DMN‐related dysfunctional manifestations in SAD patients.

#### 4.2.2 FNC between DMN and VAN/DAN

The DAN includes the cerebral cortex which supports sustained voluntary orienting, and encodes neural information associated with behavioral stimuli in higher‐order cognitive tasks^77^; the VAN, along with the DAN, contributes to the orientation of stimulus‐driven attention and automatic orienting to a stimulus location.^78^ Generally, the task‐positive DAN and VAN help manage rules and goals in the context of externally directed tasks,^79^ while the task‐negative DMN deactivates during goal‐directed behavior with focused attention.^70^ These systems work competitively, switching between internally and externally oriented cognitive processing,^80^ their proper interaction being critical for emotional regulation, cognitive control, and episodic memory performance.^81^, ^82^ As a result, our observation of decreased FNC between aDMN and DAN/VAN may be evidence of disrupted switching between internally and externally oriented cognitive control and emotional regulation in SAD.

#### 4.2.3 FNC between aDMN and perceptual systems

We observed decreased FNC between aDMN (a higher‐order cognitive control system) and the low‐level perceptual system represented by dSMN, lVN, pVN, and AUN in SAD patients. Persistent cognitive biases concerning socially threatening cues, especially voices and faces, are reported in SAD patients.^83^ The perceptual system is involved in information transmission in the context of the external environment, emotion perception and experience,^84^ perceptions of facial fear expression (for processing of social facial information),^85^ and emotional regulation in the face of threatening signals.^86^ Further, audiovisual integration requires coordination of feedback information flow among higher‐order cognitive systems and primary perceptual networks.^87^ Consequently, dysfunctional integration of audiovisual information in corresponding perceptual networks and disturbed cognitive modulation in the higher integrative networks may cause incongruous processing of external social signals, aberrant emotional arousal, and biased cognition.^88^, ^89^ In such light, one could speculate that decreased FNC among the higher‐order cognitive control and perceptual networks may reflect disrupted dynamic reconfiguration of perceptual networks and a failed dynamic integration of higher‐order processes, which may be responsible for hyperarousal/hypervigilance toward socially threatening stimuli, persistent heightened attentiveness and biased processing for sensory information, and disrupted cognitive control in patients with SAD.^90^

### 4.3 Limitations and future directions

This study has some limitations. First, we did not measure general intelligence or similar indices for the match with HC. However, there is no empirical evidence of intellectual impairment in SAD patients,^1^ which limits the potential confounding effects of general intelligence on our results. Second, this cross‐sectional study cannot inform direct causal relations between aberrant dFNC patterns and disease. This will require longitudinal studies both on SAD patients and those at high risk of developing it (e.g., based on genotypes and endophenotypes).^91^ Third, when recruiting participants, we used power analysis to confirm adequate statistical power, but our sample is still smaller than some recent neuroimaging studies on other psychiatric disorders. A main reason for this is that we adopted strict inclusion criteria, only recruiting adult SAD patients with no psychiatric comorbidity; this enabled us to explore the pure neurobiological substrates of SAD, but at some cost to the generalizability of our findings. This merits further studies with a larger sample to investigate the potential effects of demographic confounding factors on dFNC. Fourth, although we adopted the widely used and well‐established sliding‐window method to characterize dFNC patterns, how to define reasonable and optimal connectivity states is still an open (even controversial) issue.^32^, ^92^ Identifying dFNC states with more advanced approaches may be beneficial both for the development of this field and the validation of our observations.

## 5 CONCLUSION

Using a data‐driven ICA and sliding‐window approach with a k‐means clustering algorithm, this study identified four intrinsic dFNC states in the whole rs‐fMRI scans, in which SAD patients had higher occurrence and mean dwelling time in a weakly connected state but spent less time in strongly connected states; SAD patients demonstrated abnormal functional interactions across the high‐order cognitive networks and the primary perceptual systems, some of which were related to illness duration. In line with the aim of psychoradiology,^93^, ^94^ our study could offer a new insight into the dynamic neural mechanisms underlying SAD, which may facilitate the identification of clinically useful neuro‐functional biomarkers.

## AUTHOR CONTRIBUTIONS

QYG supervised the conduct of the study. QYG and XZ conceptualized and designed the study. XZ and XY contributed to the data collection. BLW provided methodological advice. XZ performed the data analysis, results interpretation and visualization, original draft writing, and editing. SW and GJK provided interpretive advice and critically revised the manuscript, which all authors reviewed and approved for publication.

## CONFLICT OF INTEREST STATEMENT

The authors declare no conflict of interest.

## Supporting information


Figures S1–S4.

## Data Availability

The data and code that support the findings of the present study are available from the corresponding author through reasonable request. The data and code sharing adopted by the authors comply with the requirements of the funding institute and with institutional ethics approval.
