# Supplementary material for: Abnormal large‐scale brain functional network dynamics in social anxiety disorder
Source: CNS Neurosci Ther. 2024 Aug 6;30(8):e14904. doi: 10.1111/cns.14904 (PMC11303268; doi:10.1111/cns.14904)

# Online Supplementary Materials

**Abnormal large-scale brain functional network dynamics in social anxiety disorder**

#### Supplementary Figures

**Figure S1.** **Spatial maps of 15 selected independent components.** Each panel shows three anatomical views of one network. Abbreviations: aDMN, anterior default mode network; aSN, anterior salience network; AUN, auditory network; DAN, dorsal attention network; dSMN, dorsal sensorimotor network; lFPN, left frontoparietal network; lVN, lateral visual network; mVN, medial visual network; pDMN, posterior default mode network; pSN, posterior salience network; pVN, posterior visual network; rFPN, right frontoparietal network; SCN, subcortical network; VAN, ventral attention network; vSMN, ventral sensorimotor network.


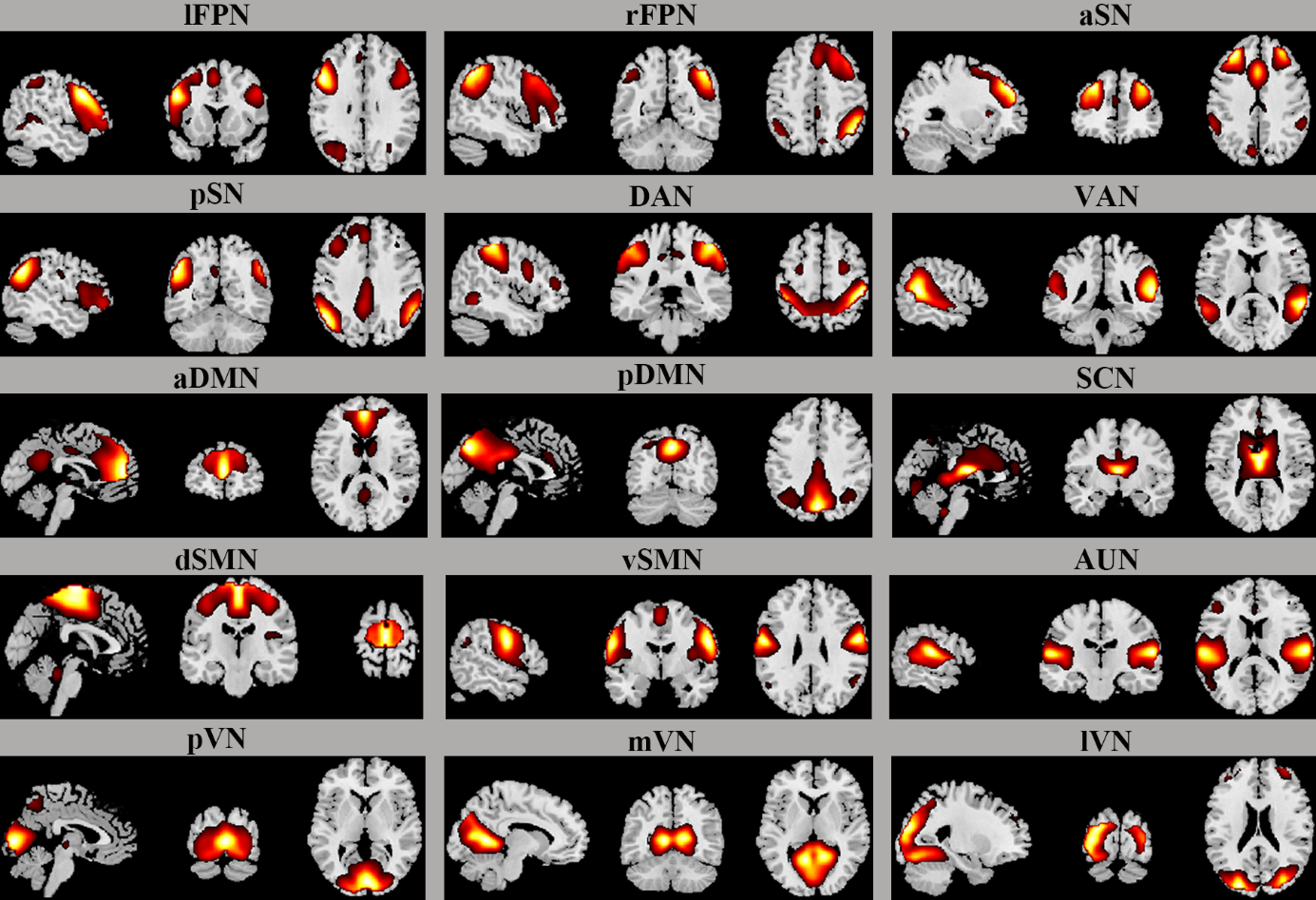


**Figure S2. Connections with the top 20% in functional network connectivity strength for each state over the whole sample (window size: 22-TR).** (A) Connections with the top 20% in functional network connectivity strength of corresponding cluster centroids. (B) Radar map delineates the mean functional network connectivity strength of each of the 15 networks for each of the 4 states. Abbreviations: aDMN, anterior default mode network; aSN, anterior salience network; AUN, auditory network; DAN, dorsal attention network; dSMN, dorsal sensorimotor network; lFPN, left frontoparietal network; lVN, lateral visual network; mVN, medial visual network; pDMN, posterior default mode network; pSN, posterior salience network; pVN, posterior visual network; rFPN, right frontoparietal network; SCN, subcortical network; VAN, ventral attention network; vSMN, ventral sensorimotor network.


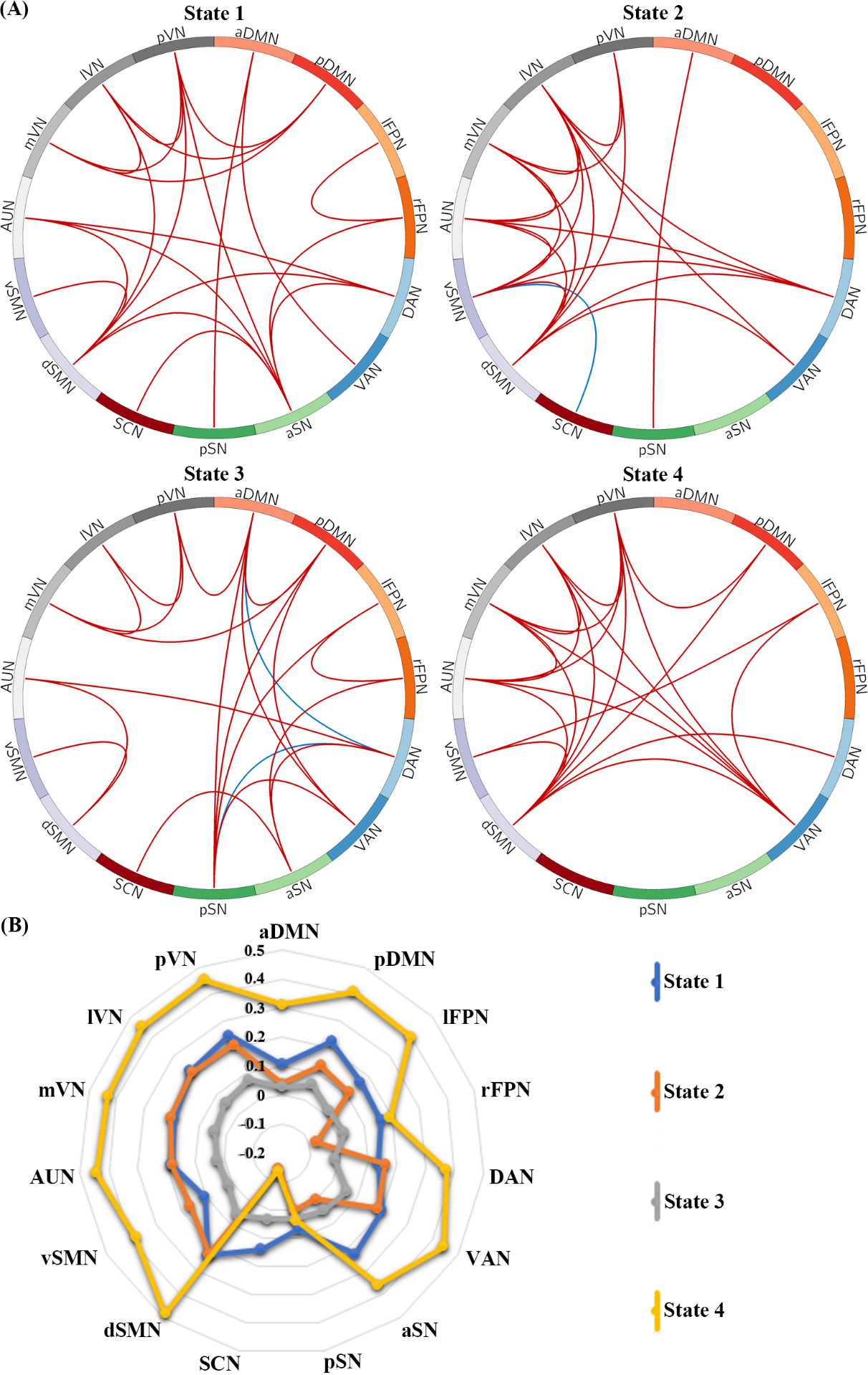


**Figure S3. Cluster centroids for each state and between-group differences of temporal transition vectors in the validation analysis with the window size set to 20-TR.** * *P* < 0.05 for group comparisons between SAD patients and HC. Abbreviations: aDMN, anterior default mode network; aSN, anterior salience network; AUN, auditory network; DAN, dorsal attention network; dSMN, dorsal sensorimotor network; HC, healthy controls; lFPN, left frontoparietal network; lVN, lateral visual network; mVN, medial visual network; pDMN, posterior default mode network; pSN, posterior salience network; pVN, posterior visual network; rFPN, right frontoparietal network; SAD, social anxiety disorder; SCN, subcortical network; VAN, ventral attention network; vSMN, ventral sensorimotor network.


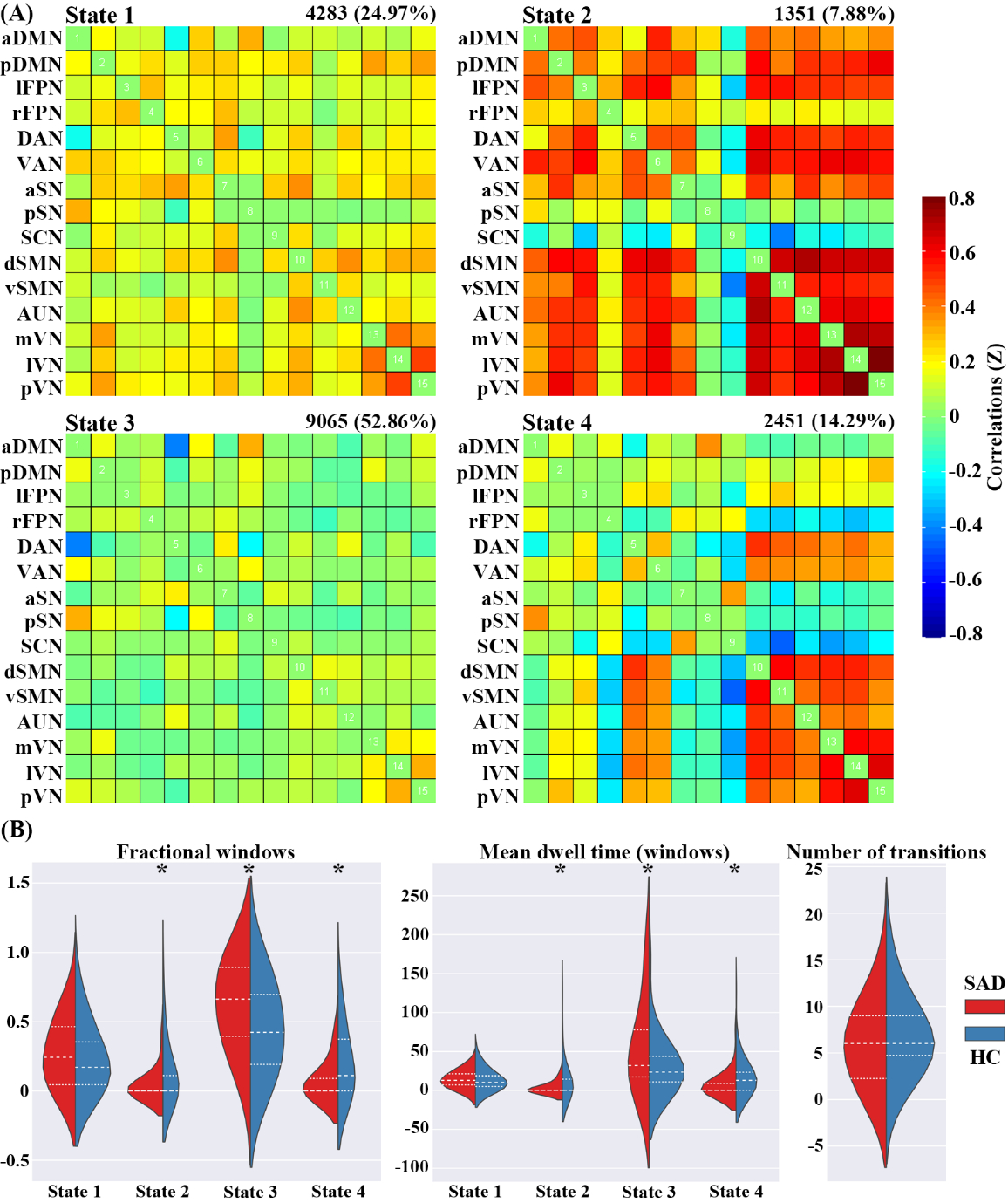


**Figure S4. Cluster centroids for each state and between-group differences of temporal transition vectors in the validation analysis with the window size set to 30-TR.** * *P* < 0.05 for group comparisons between SAD patients and HC. Abbreviations: aDMN, anterior default mode network; aSN, anterior salience network; AUN, auditory network; DAN, dorsal attention network; dSMN, dorsal sensorimotor network; HC, healthy controls; lFPN, left frontoparietal network; lVN, lateral visual network; mVN, medial visual network; pDMN, posterior default mode network; pSN, posterior salience network; pVN, posterior visual network; rFPN, right frontoparietal network; SAD, social anxiety disorder; SCN, subcortical network; VAN, ventral attention network; vSMN, ventral sensorimotor network.


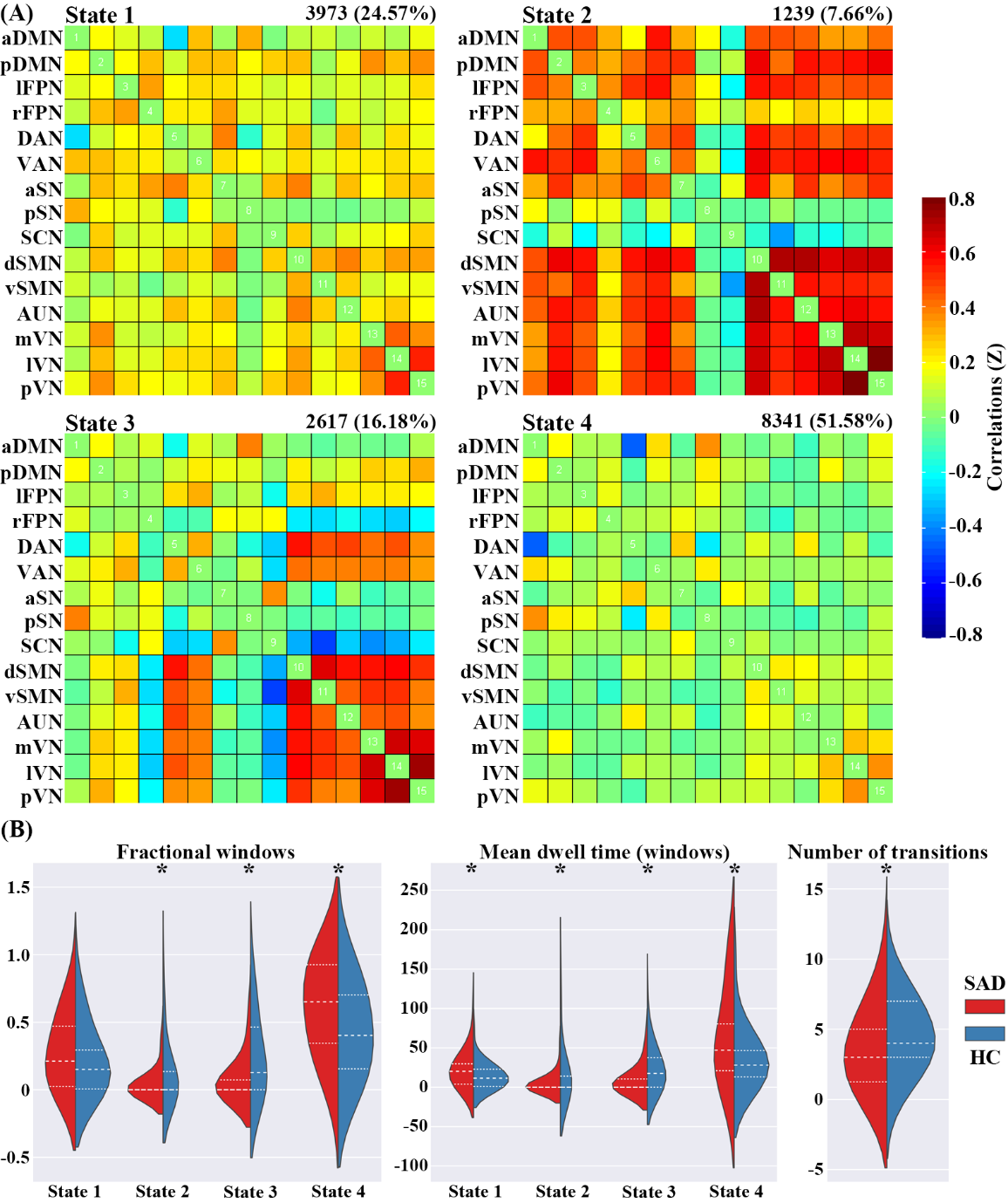

Supplement: Supplementary file 1 — Figures S1–S4. [file CNS-30-e14904-s001.docx]
